# Moving from Rabies Research to Rabies Control: Lessons from India

**DOI:** 10.1371/journal.pntd.0001748

**Published:** 2012-08-07

**Authors:** Manish Kakkar, Vidya Venkataramanan, Sampath Krishnan, Ritu Singh Chauhan, Syed Shahid Abbas, on behalf of Roadmap to Combat Zoonoses in India (RCZI) initiative

**Affiliations:** 1 Public Health Foundation of India, New Delhi, India; 2 Office of World Health Organization Representative to India, New Delhi, India; Centers for Disease Control and Prevention, United States of America

## Abstract

**Background:**

Despite the availability of effective interventions and public recognition of the severity of the problem, rabies continues to suffer neglect by programme planners in India and other low and middle income countries. We investigate whether this state of ‘policy impasse’ is due to, at least in part, the research community not catering to the information needs of the policy makers.

**Methods & Findings:**

Our objective was to review the research output on rabies from India and examine its alignment with national policy priorities. A systematic literature review of all rabies research articles published from India between 2001 and 2011 was conducted. The distribution of conducted research was compared to the findings of an earlier research prioritization exercise. It was found that a total of 93 research articles were published from India since 2001, out of which 61% consisted of laboratory based studies focussing on rabies virus. Animals were the least studied group, comprising only 8% of the research output. One third of the articles were published in three journals focussing on vaccines and infectious disease epidemiology and the top 4 institutions (2 each from the animal and human health sectors) collectively produced 49% of the national research output. Biomedical research related to development of new interventions dominated the total output as opposed to the identified priority domains of socio-politic-economic research, basic epidemiological research and research to improve existing interventions.

**Conclusion:**

The paper highlights the gaps between rabies research and policy needs, and makes the case for developing a strategic research agenda that focusses on rabies control as an expected outcome.

## Author Summary

Rabies is among the most widely spread zoonoses (diseases that are naturally transmitted between vertebrate animals and humans) in humans in most Asian, African and Latin American countries. Even though researchers have demonstrated effectiveness of strategies to control rabies at the population level, such as post exposure prophylaxis in humans and animal birth control and immunization among dogs, are well known, policy makers in most countries are hesitant to implement these strategies. This paper examines the disconnect that prevents the translation of scientific research outputs into effective policies. We contrasted the type of research papers published on rabies from India in the last eleven years with a previously identified set of priority research options. We found that most published research articles related to biomedical research focussing on development of new interventions. This was in contrast to policy and systems-related research and research to improve the performance of existing interventions that were identified as priority research options for India earlier. The findings of our study highlight the importance of moving beyond a purely researcher-driven agenda and suggest the need to promote research that has a vision of rabies control in the near future.

## Introduction

South Asian countries contribute to more than half of the global burden of rabies [1], [2]. However, in spite of the long-standing nature of the problem, and despite the presence of effective intervention strategies [3] for rabies control, rabies continues to pose a major public health challenge to program planners in the region and elsewhere. Most South Asian countries still retain ad hoc approaches and have not been able to develop sustainable, population-level rabies control strategies, such as routine availability of post exposure prophylaxis in humans, dog immunization and dog population control [4]–[6].

As demonstrated in Africa, doubts persist among some experts as well as policy makers in low resource settings regarding the technical and operational challenges of rabies control [7]. Concerns related to burden and distribution of rabies as well as cost effectiveness and practicality of the interventions persist among opinion makers even in the face of proven intervention strategies across multiple settings [7].

We propose that this state of ‘policy impasse’ is contributed by the fact, at least in part, that the research community has not catered to the information needs of the policy makers. This phenomenon is not exclusive to rabies. In fact, research to implementation gap has been reported in many other health domains [8] where the mismatch between the outputs from researchers and policy makers' information needs have been described as a key barrier to bridging this gap [9].

India is a major contributor to the global rabies burden, being responsible for 17,000–20,000 of the 55,000–70,000 deaths that modelling approaches have suggested to occur globally each year [1], [2]. In addition, the country has strong institutional capacity for research in medical, veterinary medicine and laboratory sciences.

An earlier research prioritization exercise systematically identified priority research options required for prevention and control of zoonoses in India over the next five years (2010–15) and incorporated the perspectives of a diverse group of stakeholders [10]. Rabies was also specifically identified as a priority zoonosis for India. The exercise found that the identified priority research options highlighted the importance of ‘actionable policy-relevant research’ for the prevention and control of zoonoses in India. The priorities cut across diseases, disciplines, and sectors and focussed more on policy relevant research than research for development of newer biomedical interventions.

In this paper, we build upon the findings of the earlier study to systematically review the rabies research output from India and examine its alignment with policy priorities of the country. This review is intended to serve as a case study highlighting the research – policy gap related to rabies in low and middle income countries (LMICs).

## Methods

### Search Strategy, Screening and Inclusion

The study was designed as a review of rabies-related research published from Indian institutions from 2001 to 2011 as indexed in the PubMed database. PubMed was selected for the search as it is among the most accessible, standardized and extensive sources of life sciences literature in India, covering research publications in veterinary sciences, public health and molecular biology.

The search was restricted to Indian institutions publishing rabies research since 2001 so as to ascertain the national research capacity and its alignment with national policy needs as reflected in the prioritisation exercise referred to earlier [10]. We aimed to employ an inclusive search strategy to ensure maximum coverage of original research related to rabies from India. The following search terms were used: *“rabies”[MeSH Terms] OR “rabies”[All Fields]) AND india[Affiliation] AND (“2001/01/01”[PDat] : “2011/31/12”[PDat]*.

All original research articles related to rabies published from an Indian institution were included in the review. Articles not related to rabies as an important focus area of the study, case studies, literature reviews, opinion pieces and meeting reports were excluded. The review assessed the concordance between conducted research and policy priorities. Given the topical nature of policy agendas, the review was confined to research conducted in the last eleven years so that these could be contrasted with contemporary policy priorities.

A total of 138 articles related to rabies were identified to have been published from India in the last eleven years through PubMed. An initial screening of the records resulted in the exclusion of one PubMed reference to an erratum. Subsequently, the remaining 137 articles were reviewed by two researchers for inclusion in the final database using the criteria described above. Any conflicts in the process were resolved through mutual discussions or consultation with a third researcher.

### Data Extraction

Once the list of articles was finalized, their abstracts were reviewed for extracting metadata on publishing journal, setting of research and institutional affiliation of researchers. The articles were then categorized into research categories used in an earlier research prioritization exercise for zoonoses prevention and control: Instruments of Health Research (IHR) and Research Factorials [10]. While the IHR [11] aimed to assess the actionable nature of the findings expected from the research question, the research factorial categories [12] sought to assess the involvement of different sectors in the research question. A listing of these categorizations is mentioned in Table 1. Detailed descriptions of the research categorizations have been included as a supporting file (File S1). The process of categorization was carried out primarily by one researcher and a sample of categorizations was reviewed by the second researcher. Any confusions relating to the categorizations or conflict between the ratings of the two researchers were highlighted and resolved through mutual discussion with a third researcher. The proportionate distribution of conducted research into IHRs and the research factorial categories was then compared to their distribution in priority research options identified earlier by national experts and policy makers [10].

**Table 1:** Proportional distribution of rabies research articles in India across research categories.

Category	No. of Articles	%
**Journal Type**		
**General**	45	48%
**Human**	40	43%
**Animal**	7	8%
**Plants**	1	1%
**Sector**		
**Human**	53	57%
**Animal**	27	29%
**General**	13	14%
**Plant**	0	0%
**Environment**	0	0%
**Setting**		
**Lab-based**	57	61%
**Clinical/Facility-based**	25	27%
**Urban community-based**	5	5%
**Urban-Rural (mixed) community-based**	3	3%
**Occupational**	2	2%
**Rural community-based**	1	1%
**Species**		
**Pathogen**	54	58%
**Human**	32	34%
**Dog**	6	6%
**Multiple Animals**	1	1%
**Plants**	0	0%
**Instruments of Health Research**		
**Research for development of new interventions**	58	62%
**Basic Epidemiologic Research**	26	28%
**Research to improve existing interventions**	6	6%
**Health policy and systems research**	3	3%
**Factorial**		
**Genetic and Biological**	81	87%
**Social, Political, Economic (including Epidemiology)**	12	13%
**Physical and Environmental**	0	0%
**Ecological**	0	0%
**Grand Total**	**93**	**100%**

## Results

**Figure 1:**
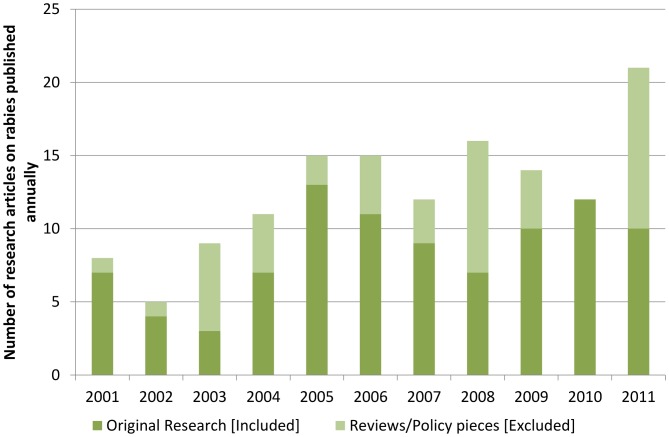
Annual research output related to rabies from India (2001–11).

A total of 138 rabies-related publications were identified from India, which represents 4.4% of the total global research output on rabies in the same period (3,113 articles). Approximately 33% of the identified abstracts were excluded as they were review pieces or not directly related to rabies. A total of 93 original research articles were identified for detailed categorizations. On average, 8.5 original research articles on rabies were published from India every year and, as depicted in Figure 1, the number of research papers published per year varied from 3 to 13.

The distribution of rabies research output based upon the research categorizations is described in detail below; Table 1 summarises the key findings. The full list of articles included for analysis along with their categorizations is included as supporting file (File S2).

### Journals

Journals focussing on animal health accounted for only 8% of the publications. Most of the articles were published in broad-based or human centric journals (48% and 43%, respectively). The 93 identified articles were published through 50 different journals. However, the top three journals accounted for 30% of all the published articles. These journals were *Vaccine, Human Vaccines* and *International Journal of Infectious Diseases*.

### Research Institutions

Institutions having Ministry of Health & Family Welfare as the nodal ministry dominated rabies research output, accounting for 57% of identified articles. The veterinary sector followed with 27%, and other institutions contributed 14% of publications. The top two institutions from the human and animal health sectors together accounted for half the total research output. These were National Institute of Mental Health & Neuro Sciences (NIMHANS), Kempegowda Institute of Medical Sciences (KIMS), Indian Veterinary Research Institute (IVRI) and Indian Immunologicals Limited (IIL).

We identified a total of 29 institutions from human, veterinary and other sectors that have worked on rabies research in the last eleven years. While half (14) the institutions were from the health sector, one third (9) were from the veterinary sector. The category of other institutions (6) included those from the scientific institutions and the vaccine industry.

### Settings & Species

The vast majority of published articles (58%) related to the rabies virus and a third (34%) were human-focussed. Only a minority of articles focussed on dogs (7%) and other animals (1%). The predominantly bio-medical focus of rabies research was also borne out by a categorisation of settings in which the reported research took place. While 61% of research articles described laboratory based work, 27% of articles related to clinic based research. Only 12% of research articles related to community based research settings. This trend was more pronounced for the veterinary sector where 23 out of a total of 25 articles related to laboratory based research. In contrast, research in the human sector was almost evenly divided between clinical and laboratory research.

### Type of Research

**Figure 2:**
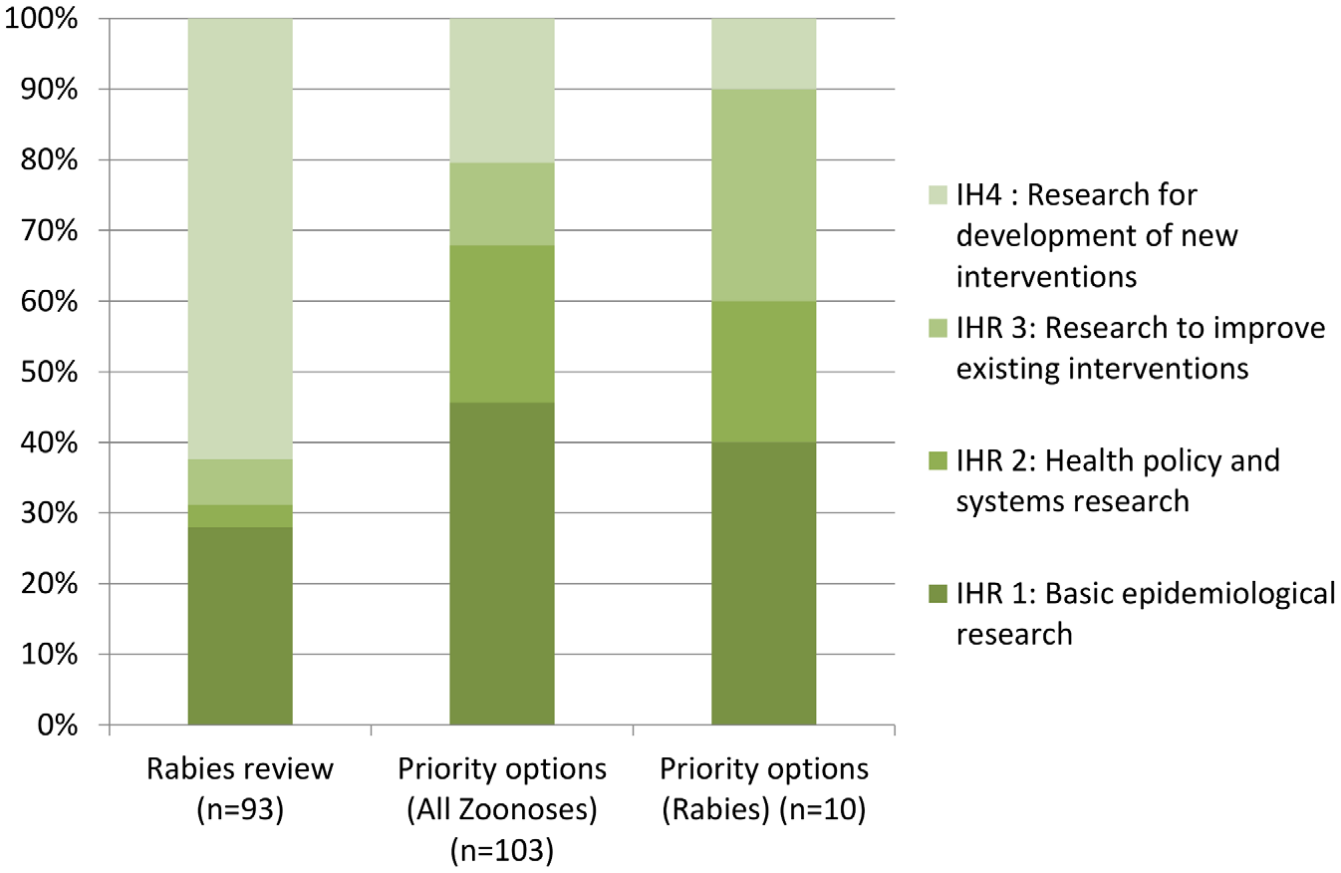
Rabies research output, categorized by Instruments of Health Research [**10**].

As described in Table 1 and Figure 2, a large proportion of rabies research related to basic science research for the development of new interventions. Most of the remaining research options related to epidemiologic research. Less than 10% of conducted research related to improving existing interventions or for research related to health policy and systems.

**Figure 3:**
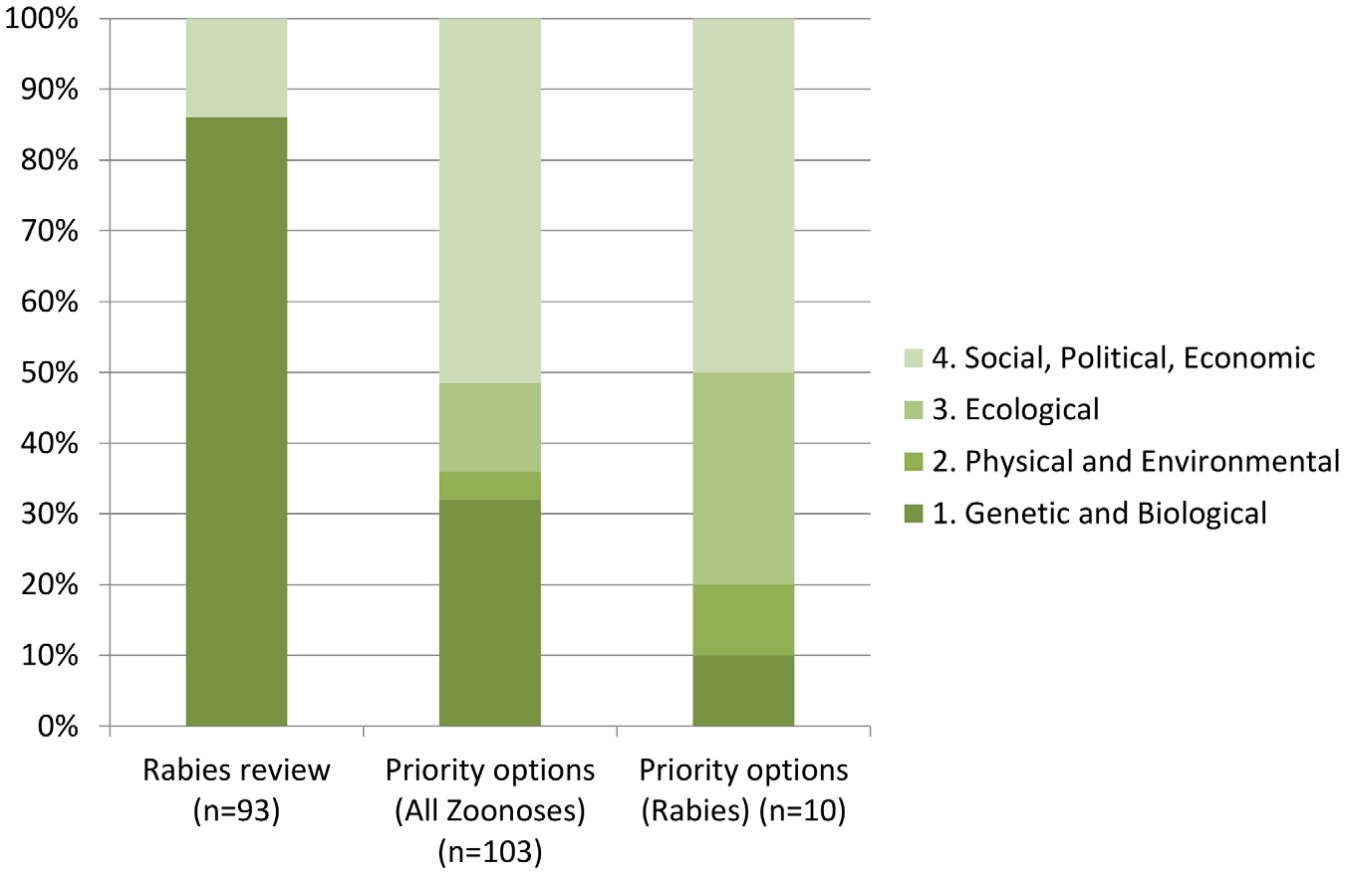
Rabies research output, categorized by research factorials [**12**].

Adopting a different lens of research factorials (Figure 3), we found that the research conducted so far was almost entirely focussed upon genetic and biological factors (86%) and some social, political and economic research (14%).

## Discussion

Researchers have highlighted the importance of periodically reviewing health research for its relevance to policy requirements in multiple contexts [11], [13], [14]. This is especially true in case of neglected diseases, where the cause of neglect could very well be because of the absence of policy-relevant research [15]. It is therefore important to compare the conducted research with policy requirements as a component of priority setting exercises. This review is the first of its kind that seeks to trace the research-policy gaps for rabies control by reviewing conducted research and contrasting it with priority research areas.

### Research Output

Research output in terms of number of articles was fairly regular over an eleven year period, averaging 8.5 research articles per year. However, given the fact that India contributes less than 5% of global research output on rabies yet contains half the disease burden, the quantum of research output does not appear to be in keeping with either the disease burden in India or its institutional research capacity.

Research priorities were clearly skewed towards a bio-medical disease paradigm, with pathogen-based research driving the research agenda. Laboratory -based and clinical research focussing on the virus and its disease manifestations appeared to be more popular than risk research, ecological studies, health services research, operations research, economic evaluations and health systems research. As demonstrated by other researchers, this phenomenon is not limited to rabies and is a reflection of limited focus on public health research in India [13], [16], [17] and globally [18]–[20].

### Priority Research Areas Vs. Conducted Research

The distribution pattern of conducted research topics on rabies appeared to be in direct contrast with the research options identified by national experts and policy makers in an earlier study [10]. (The list of priority research options related to rabies control in India can be found in File S3.) As described in Figure 2, the priority research options for all zoonoses (n = 103) as well as priority research options specifically for rabies (n = 10) had a much more balanced distribution of IHRs and research factorials than what was found among the conducted research. Research for development of new interventions was least favoured among priority research options, while it was the most represented research option among the review articles.

Similarly, as depicted in Figure 3, the distribution pattern of research factors in the review articles was inverse of what was found in the priority research options. The priority research options also focussed upon ecological, physical and environmental factors that were totally absent from the conducted research.

While the importance of strengthening basic science research in the long run cannot be disputed, it needs to be understood that program managers and policy makers operate on shorter time frames than researchers. They need more actionable information from the research community, a role that can easily be fulfilled by the public health and veterinary community conversant with the practical challenges of mounting rabies intervention strategies.

### Research–Policy Gap

It is important to situate the findings from our review into the larger context of policy challenges facing rabies control in India and internationally. In spite of repeated attempts, efforts to create a national rabies control program in many LMICs, including India, have not been successful because of challenges in conceptualising a programmatic structure for a multisectoral effort. A national consultation of rabies researchers, program managers and policy makers organised recently in Chennai, India, reviewed the policy landscape of rabies control in India and recognised the fact that rabies-related policy making has largely been conducted in isolation, with little contribution from local research [21].

Inadequate interaction and communication between the research and policy-making communities is caused and exacerbated by the lack of collaborative platforms, differences in perspectives, and institutional barriers. Researchers are often unaware about the information needs of policy makers, while policy makers face limitations in preparing evidence-informed policies.

We describe some of the key policy challenges facing rabies planners in India and a sample of indicative knowledge gaps relating to these issues in Table 2. The identified knowledge gaps are of immediate relevance to policy makers, and filling these gaps can lead to the development of national implementation framework for rabies control in India. Unfortunately, we were unable to find much conducted research that could help answer these questions.

**Table 2:** An illustrative list of knowledge gaps derived from rabies policy challenges.

Policy & Program issue	Example	Indicative knowledge gap	Relevance for program planners
**Intersectoral Coordination**	No national programme in spite of high disease burden and repeated efforts, due to lack of clarity on role of animal husbandry department [29].	What are the contours of coordination mechanisms that facilitate joint planning and implementation of interventions?	Enable effective and efficient use of resources to implement joint rabies interventions; will also provide a template to tackle other zoonotic diseases, including EIDs.
**Scale-up and Replicability**	Successful pilot intervention for rabies control in localised urban settings could not be replicated at state/national level [30], [31].	What are the factors that will allow replication and scaling up of successful pilot interventions?	Institute mechanisms to implement state/national level rabies control strategies.
**Census**	Widely varying dog population estimates across consecutive rounds of census; limited information on dog ecology [32]–[34].	How do dog population groups respond to different sets of intervention strategies?	Guide dog population management.
**Surveillance**	Poor quality of surveillance data both from human and animal sides, wherever reported [6], [35], [36].	What are the more pragmatic surveillance standards to improve the coverage and quality of surveillance systems for rabies among humans and animals?	Promote evidence based planning and evaluation.
**Vaccine requirement**	Limited data on dog bite epidemiology for predicting vaccine requirements [37].	What is the caseload of severe dog bite cases at different levels of health facilities?	Enable estimation of vaccine and antibody requirements.
**Diagnostics**	Weak diagnostic capacity [21], [38].	What are the barriers to establishing a rabies diagnosis network?	Provide capacity to improve surveillance quality and address underdiagnoses.
**Impact of Interventions**	Limited evidence on efficacy and effectiveness of interventions in different ecological settings.	What set of interventions will work at the population level in different parts of the country?	Allow long term planning and resource allocation for appropriate mix of rabies interventions strategies.
**Environmental management**	Lack of thrust on environmental management in rabies control strategies [39].	What could be the potential intervention strategies of environmental management for rabies control?	Contribute to dog population management.

### Developing a Research Agenda for Rabies Control

While rabies research in India might not be completely reflective of global priorities, we have used it as an illustrative case study to highlight points that can be used to inform a larger discussion on prioritisation for rabies research globally. Researchers have reported similar research-policy disconnect in rabies control in other Asian and African countries. Their concerns relate to the absence of political commitment for rabies control from decision makers as a result of a perceived lack of conclusive information on disease burden and cost effectiveness of existing interventions among others [4], [5], [7].

In order to overcome this stalemate and ensure progressive action towards rabies control globally, we propose the development of a strategic research agenda at national and regional levels *focussing on rabies control* among affected populations as an expected outcome. Such a research agenda would help the planners evolve a unified vision of rabies control involving a closer interaction of different *disciplines* (epidemiology, economics, life sciences and sociology, among others), *sectors* (human, animal and environment) as well as *functions* (researchers, practitioners, policy planners, donor representatives). Existing frameworks on national research systems [22] and zoonotic research [23] can be used to inform the development of such a strategic research agenda for combating rabies at the national and regional levels.

The policy relevance of conducted research can increase only when the close relationships between policy, program and research functions are recognized and when both research generators as well as research users are equally invested in such an exercise. Development of such a research agenda should, therefore, involve all stakeholder communities for a series of exercises going beyond defining the research needs of a specific population group. While the stewardship and the larger vision will need to come from the policy makers and nodal agencies with the mandate to lead such efforts, the researcher and program manager communities will need to be mobilised to advocate for the development and implementation of such an agenda.

As a first step, periodic research prioritization exercises will play a necessary role in aligning research output to the public health needs of the community. Recent initiatives on research prioritization have demonstrated the importance of increasing the policy relevance of conducted research across specific areas [11], institutions [24] and national research systems [25]. The research agenda will also need to include other mechanisms to increase knowledge translation [26] processes. An indicative list of mechanisms for promoting research-policy interactions include the following: creating knowledge networks, establishing partnerships and allowing mutual exchange of personnel between research, training and implementing organizations, increasing emphasis on evidence based decision making, creating information clearing house, etc [9], [27].

### Study Limitations

Possible limitations in the study design that may affect the robustness of its results and the generalizability of its conclusions are listed below.

First, we restricted our search to PubMed because of ease of search and the database' coverage of multiple sectors. Although “grey literature” and un-indexed papers were not included as a result of this strategy, PubMed has the largest coverage of all life sciences journals, which ensures that we have captured the majority of literature on rabies research.

Second, the corresponding author affiliation was the only way to capture national affiliations. It is possible that resident researchers would have conducted policy-relevant research in collaboration with non-Indian institutions, but we did not include this work as it was not seen to be contributing to the capacity of national institutional research.

Third, we have referred to an earlier priority setting exercise by our team that can be used as a comparison with the conducted research. We would have liked to include further measures for validating our conclusions, but to our knowledge, we are not aware of other systematic priority setting exercises in zoonoses in this region. The purpose of this paper was to highlight the need for strategic planning of rabies research and to identify key issues that should be considered in the process using the example of India. The exact processes involved and the identification of precise criteria for research prioritizations will have to be informed by the local context.

### Conclusions

Rabies research globally has generated a lot of ‘actionable’ evidence related to rabies control. Yet rabies control efforts continue to be neglected in many LMICs. We use the example of rabies research in India to demonstrate the fact that the research community has not been able to sufficiently address the concerns of policymakers. While the rabies research output in India is neither reflective of its share of the disease burden nor its institutional capacity, rabies research conducted in India has the potential to influence the rabies agenda nationally as well as in many LMIC countries if more policy relevant research is conducted.

The Planning Commission, Government of India has identified rabies as a priority zoonosis in India that will be targeted through a set of focussed strategies [28]. However, there is no strong evidence base to appropriately inform this well-intentioned strategy. There is an urgent need to address this research-policy gap by developing a strategic research agenda for rabies control at the national and regional levels. Our observations on rabies research in India can be used as a predictor of similar challenges in other LMICs. Therefore, we contend that program priorities should be an important factor in systematically shaping research agendas related to rabies in India and other endemic countries.

## Supporting Information

File S1 **Categorizations and definitions.**
(DOC)

File S2 **List of rabies research articles from India (2001–11).**
(XLS)

File S3 **List of priority research options for rabies.**
(DOC)
