# Supplementary material for: Moving from Rabies Research to Rabies Control: Lessons from India
Source: PLoS Negl Trop Dis. 2012 Aug 7;6(8):e1748. doi: 10.1371/journal.pntd.0001748 (PMC3413711; doi:10.1371/journal.pntd.0001748)
Supplement: File S1 — Categorizations and definitions. (DOC) [file pntd.0001748.s001.doc]

# Supporting file S1: Categorizations and Definitions

| **Categorization** | **Research categories** | **Description of research categories** |
| --- | --- | --- |
| **Instruments of Health Research [1,2]** | IHR1. Basic Epidemiologic Research | *defines disease burden, its components, relative risks of different underlying factors and efficacy of the available interventions to reduce the burden; includes*  *A. Measuring the burden*  *B. Understanding risk factors*  *C. Evaluating existing interventions* |
|  | IHR2. Health policy and systems research | generates new knowledge to enable more efficient use of available health care resources in reducing disease burden; includes  D. Studying system capacity to reduce exposure to proven health risks  E. Studying system capacity to deliver efficacious interventions |
|  | IHR3. Research to improve existing interventions | *aims to improve deliverability, affordability and sustainability of those interventions of proven efficacy; includes*  *F. Research to improve deliverability of existing interventions*  *G. Research to improve affordability of existing interventions*  *H. Research to improve sustainability of existing interventions* |
|  | IHR4. Research for development of new interventions | *includes approaches that leads to development of new interventions, ranging from exploring the role of possible risk factors to basic molecular and genomic research that would help understand processes leading to zoonotic disease; includes*  *I. Basic research*  *J. Clinical research*  *K. Public health research* |
|  |  |  |
| **Research Factorial [1,3]** | F1. Genetic and Biological | *Examples: microbial adaptation and change; human susceptibility to infection* |
|  | F2. Physical and Environmental | *Examples: climate and weather patterns; physical events (e.g., earthquakes, flooding)* |
|  | F3. Ecological | *Examples: changing ecosystems; development and land use* |
|  | F4. Social, Political, Economic | *Examples: human demographics and behavior; technology and industry; travel and commerce; poverty and social inequity; war and famine; bioterrorism or intent to harm; lack of political will; breakdown of public health measures* |
|  |  |  |
| **Journal Type** | J1. Human | Journals that publish studies on or directly related to humans, as stated on their website |
|  | J2. Animal | Journals that publish studies on or directly related to animals, as stated on their website |
|  | J3. Plants | Journals that publish studies on or directly related to plants, as stated on their website |
|  | J4. General | Journals that publish studies on thematic areas cutting across species, as stated on their website |
|  |  |  |
| **Setting** | SET1. Urban community-based | Research where principle amount of data collection has been undertaken within urban community settings; E.g. all kinds of surveys and epidemiologic studies located in urban areas among urban populations |
|  | SET2. Rural community-based | Research where principle amount of data collection has been undertaken within rural community settings; E.g. all kinds of surveys and epidemiologic studies among rural populations |
|  | SET3. Urban-Rural (mixed) community-based | Research where principle amount of data collection has been undertaken within community settings; E.g. all kinds of surveys and epidemiologic studies among a mix of urban-rural populations |
|  | SET4. Occupational | Research where principle amount of data collection has been undertaken within occupational settings; E.g. all kinds of surveys and epidemiologic studies located in industrial areas or among specific occupational groups |
|  | SET5. Clinical/Facility-based | Research where principle amount of data collection and analysis have been conducted within a clinical facility (humans as well as animals); generally with a patient centric approach. E.g. case reports, clinical trials |
|  |  |  |
| **Sector** | SEC1. Human | Research conducted in public or non-profit institutions that come under the purview of Ministry of Health & Family Welfare |
|  | SEC2. Animal | Research conducted in public or non-profit institutions that come under the purview of Department of Animal Husbandry, Dairying & Fisheries under Ministry of Agriculture |
|  | SEC3. General | Research conducted in institutions in Departments and Ministries other than those specified above (mostly consisting of Universities, specialised research institutions under the Ministries of Human Resource Development and Science & Technology, respectively and the corporate sector |
|  | SEC4. Plants | Research conducted in public or non-profit institutions that focus on plant based research |
|  | SEC5. Environment | Research conducted in public or non-profit institutions that focus on environmental research, including wild life |
|  |  |  |
| **Species** | SPEC1. Human | Research largely conducted on human subjects for understanding disease pattern among humans |
|  | SPEC 2. Dog | Research largely conducted on dogs for the purpose of understanding disease patterns among dogs |
|  | SPEC 3. Multiple Animals | Research involving multiple animals for the purpose of understanding disease patterns more than one animal species |
|  | SPEC 4. Pathogen | Research conducted on rabies virus for the purpose of understanding the behaviour of virus in isolation; or for the purpose of understanding specific cellular processes with respect to the virus |
|  | SPEC5: Plant | Research largely conducted on plant species for understanding health of plants |

**References**

1. Sekar N, Shah NK, Abbas SS, Kakkar M (2011) Research Options for Controlling Zoonotic Disease in India, 2010-2015. PloS one 6: e17120. Available:http://www.pubmedcentral.nih.gov/articlerender.fcgi?artid=3045424&tool=pmcentrez&rendertype=abstract. Accessed 9 March 2011.

2. Child Health And Nutrition Research Initiative (2006) A new approach for systematic priority setting. Available:http://www.chnri.org/secured/uploads/ publications/files/0535210001249198837-604_file_PRIORITY_SETTING_.pdf.

3. Committee on Emerging Microbial Threats to Health in the 21st Century (2003) Microbial Threats to Health: Emergence, Detection, and Response. Smolinski MS, Hamburg MA, Lederberg J, editors Washington DC: National Academies Press. p. Available:http://www.nap.edu/catalog/10636.html.
