# Supplementary material for: Moving from Rabies Research to Rabies Control: Lessons from India
Source: PLoS Negl Trop Dis. 2012 Aug 7;6(8):e1748. doi: 10.1371/journal.pntd.0001748 (PMC3413711; doi:10.1371/journal.pntd.0001748)
Supplement: File S3 — List of priority research options for rabies. (DOC) [file pntd.0001748.s003.doc]

# Supporting file S3: List of priority research options for rabies

An indicative list of priority research options that could contribute towards controlling of rabies in India in the next five years is given below as identified and scored by a multidisciplinary group of experts. Further details of the methodology and research options related to other zoonoses can be found in original paper.[1]

Table S3

| **Description of option** | **Weighted score** |
| --- | --- |
| 1. Determine the availability and prescribing policies of rabies vaccine at primary health centers and private facilities | 0.9542 |
| 1. Compare existing models for the production, purchase and distribution of rabies vaccines to identify best practices | 0.8971 |
| 1. What is the effectiveness of animal birth control, culling, and vaccination approaches for rabies control? | 0.8932 |
| 1. Compare the impact of various rabies policies and control programs of different states | 0.8817 |
| 1. How can we reduce rabies transmission at domestic animal and wildlife interfaces? | 0.7861 |
| 1. Document the experiences of animal bite victims as they access health services | 0.7663 |
| 1. Test the effectiveness and deliverability of oral/edible rabies vaccine in animals and wildlife | 0.7554 |
| 1. What are policy options to co-opt the private sector for rabies vaccine delivery? | 0.7078 |
| 1. Assess wildlife as reservoirs for rabies | 0.6423 |
| 1. Measure the effect of proximity of slums, dumps, rivers and forests on rabies incidence using GIS | 0.5397 |

**Reference:**

1. Sekar N, Shah NK, Abbas SS, Kakkar M (2011) Research Options for Controlling Zoonotic Disease in India, 2010-2015. PloS one 6: e17120. doi:10.1371/journal.pone.0017120.
